# Co-composting of tail vegetable with flue-cured tobacco leaves: analysis of nitrogen transformation and estimation as a seed germination agent for halophyte

**DOI:** 10.3389/fmicb.2024.1433092

**Published:** 2024-09-04

**Authors:** Chenghao Xie, Xiao Wang, Benqiang Zhang, Jiantao Liu, Peng Zhang, Guangcai Shen, Xingsheng Yin, Decai Kong, Junjie Yang, Hui Yao, Xiangwei You, Yiqiang Li

**Affiliations:** ^1^Marine Agriculture Research Center, Tobacco Research Institute, Chinese Academy of Agricultural Sciences, Qingdao, China; ^2^National Center of Technology Innovation for Comprehensive Utilization of Saline-Alkali Land, Dongying, China; ^3^Tobacco Shandong Industrial Co., Ltd., Jinan, China; ^4^Plant Functional Component Research Center, Tobacco Research Institute, Chinese Academy of Agricultural Sciences, Qingdao, China; ^5^Tobacco Baoshan Industrial Co., Ltd., Baoshan, China

**Keywords:** organic waste, tail vegetable, wild soybean, phytotoxicity, nitrogen conversion

## Abstract

Resource utilization of tail vegetables has raised increasing concerns in the modern agriculture. However, the effect and related mechanisms of flue-cured tobacco leaves on the product quality, phytotoxicity and bacterially-mediated nitrogen (N) transformation process of tail vegetable composting were poorly understood. Amendments of high-dosed (5% and 10% w/w) tobacco leaves into the compost accelerated the heating process, prolonged the time of thermophilic stage, increased the peak temperature, thereby improving maturity and shortening composting duration. The tobacco leaf amendments at the 10% (w/w) increased the N conservation (TN and NH_4_-N content) of compost, due to the supply of N-containing nutrient and promotion of organic matter degradation by tobacco leaves. Besides, tobacco leaf amendments promoted the seed germination and root development of wild soybean, exhibiting the feasibility of composting product for promoting the growth of salt-tolerant plants, but no dose-dependent effect was found for tobacco leaf amendments. Addition of high dosed (5% and 10% w/w) tobacco leaves shifted the bacterial community towards lignocellulosic and N-fixing bacteria, contributing to increasing the compost maturity and N retention. PICRUSt 2 functional prediction revealed that N-related bacterial metabolism (i.e., hydroxylamine oxidation and denitrifying process) was enhanced in the tobacco leaf treatments, which contributed to N retention and elevated nutrient quality of composting. To the best knowledge, this was the first study to explore the effect of tobacco waste additives on the nutrient transformation and halophyte growth promotion of organic waste composting. These findings will deepen the understanding of microbially-mediated N transformation and composting processes involving flue-cured tobacco leaves.

## 1 Introduction

With the rapid improvement of people living standard, the requirements for food health and nutrition have increased. As a result, the demand for meat, eggs, milk, and fresh vegetables are constantly increasing (Jin et al., 2021). Accordingly, the scale of livestock and poultry breeding in China is expanding and standardization is strengthened. However, the related pollution prevention and resource treatment work is lagging behind. Owing to the poor post-disposal (e.g., direct return to field and incineration), the enormous quantities of agricultural organic waste (e.g., livestock manure and tail vegetables) exceeded the bearing capacity of nearby natural ecosystem, posing a serious threat to environment and human health (Xie et al., 2023). Therefore, to utilize agricultural organic waste in a recycling and green manner has become an urgent issue to be addressed (He X. et al., 2022).

Agricultural waste contained a large amount of organic matter and nutrient elements such as nitrogen, phosphorus, and potassium, which can be converted into fertilizers via various technologies (Zheng et al., 2022). Tail vegetables were commonly generated from the agricultural production. It was estimated that the generation amount of tail vegetable in China reached 384 million tons in 2021 (Qian et al., 2022). Tail vegetables, characterized as low nitrogen content and high carbon content, were mostly the root, stems and leaves after the crop harvest. Most of tail vegetables were cellulose, hemicellulose, lignin, and other organic substances that were difficult to be decomposed (Zheng et al., 2022). Besides, tail vegetables commonly carried heavy metals and pathogens, increasing the ecological risks (Hoang et al., 2022). Therefore, for resource utilization and environment protection, it was very crucial to properly treat and dispose of tail vegetables. Resource utilization technologies of tail vegetables popularized in China mainly included direct return to field, incineration and aerobic composting (Qiao et al., 2023). Compared with other treatment methods, composting was an efficient and environmentally benign technology that converted solid wastes into a safer product and/or organic fertilizer (Li et al., 2023; Qian et al., 2022). Aerobic composting could maximize the material cycle and carbon capture, posing a series of positive effects (Huang et al., 2022; Shan et al., 2021). However, there were many challenges with composting, such as large N losses and greenhouse gas emissions. N loss could not only cause atmospheric pollution, but also reduce the quality of compost product. TN amount was generally lost accompanied with N-containing gas emissions (Shan et al., 2021). Hence, mitigating N loss during the composting is particularly important to improve composting quality and reduce environmental pollution. Several studies have demonstrated that application of additives into the compost could be one of highly effective methods to reduce N loss and enhance N conservation during composting (Huang et al., 2022). Adding biochar, salts (e.g., MgCl_2_, FeSO_4_) or acid–base additives [e.g., Mg(OH)_2_, H_3_PO_4_] into compost could reduce NH_3_ emission, increase NH_4_^+^ content, and reduce TN loss (Ba et al., 2020; Ye et al., 2023). Essentially, composting was a process of degradation of organic matter under the action of microorganisms. However, microbially-mediated nutrient transformation process (especially for N conservation) in the compost and related ecological impacts have not been clearly illustrated.

Tobacco was cultivated in more than 4 million hectares of land area globally. China is one of the main tobacco growers in the world (Lisuma et al., 2020). Discarded tobacco leaf was a representative of tobacco waste. A large amount of tobacco waste with high water content were produced in farmland, and it may cause environmental pollution if it was not properly treated. Tobacco waste was not easily collected and transported, resulting in its centralized treatment was expensive. However, tobacco leaves were rich in nutrients, with protein accounting for about 10% of dry matter and minerals accounting for 5–10%, of which K accounts for 0.44–0.53% (Zittel et al., 2020). It will be beneficial to solve environmental pollution, recover parts of nutrients, and increase organic matter content in the soil, if the tobacco waste can be effectively reused (Wang et al., 2020). However, the effect of tobacco leaves on the N transformation process, product quality, and phytotoxicity of tail vegetable composting and related microbially-mediated mechanisms were poorly understood. Moreover, lack of studies addressing on the nitrogen loss issues during the tail vegetable composting has motivated the investigation of the present study.

Therefore, the flue-cured leaves of tobacco (*Nicotiana tabacum* L.) were collected aerobiotic composting was conducted using mixture of tomato tail vegetable, cattle manure and rice husk as compost raw materials, different doses of flue-cured tobacco leaves as additives. The specific objectives were (1) to investigate the variation in physicochemical properties and N-transformation process during composting amended with different doses of tobacco leaves; (2) to explore how the addition of tobacco leaves influence the bacterial community structure and metabolic functions; and (3) to evaluate the promotion efficiency of composting leachate on the seed germination of salt-tolerant halophytes. This study will offer guidelines for the development of composting technology used for resource utilization of tail vegetables and application of related composting products into promoting growth of halophytes in the salt-affected soils.

## 2 Materials and methods

### 2.1 Description of compost material

Tomato tail vegetable was collected from a conventional tomato farm located in Jimo District, Qingdao. Cattle manures were obtained from the local dairy farm in the Jimo District, Qingdao, Shandong Province, China. The husk of rice (*Oryza sativa* L.) was provided by Beisenmiao Energy Company, Henan. Ultrapure water was prepared using the Milli-Q water purification system (Advantage A10, Millipore, United States). The flue-cured leaves of tobacco (*Nicotiana tabacum* L.) were provided by Tobacco Research Institute, Chinese Academy of Agricultural Sciences, Qingdao. Prior to mixing, the rice husks were cut into 0.5 cm pieces and flue-cured tobacco leaves were passed through 2 mm sieve.

### 2.2 Composting process and sampling

Rice husk, tomato tail vegetable and cattle manure were mixed evenly at 5:4:1 (wet weight) to ensure the C/N ratio around 30. Composting in each treatment was conducted in three sealed 40 L-cylindrical rotary drum composters for 50 days. Each treatment was replicated three times. The proper amount of ultrapure water was added into the mixture in each rotary drum composter to maintain the moisture content at the 65%. Temperature was monitored every day between 9:00 and 10:00 a.m. The adding ratio of tobacco leaves with raw material mixture (RM), including 0 (%w/w), 2 (%w/w), 5 (%w/w) and 10 (%w/w). Therefore, four composting treatments were set up (1) RM: tomato tail vegetable + cattle manure + rice husk (CKT); (2) RM + 2% (w/w) tobacco leaves (LT); (3) RM + 5% (w/w) tobacco leaves (MT); (4) RM + 10% (w/w) tobacco leaves (HT). Samples weighting about 200 g were collected from each rotary drum composter after mixing thoroughly from top to bottom layers in the composters on day 3, 7, 9, 14, 28, and 50 for the analysis of physiochemical properties. During the compost sampling, all the instruments were sterilized. About 0.5 g of fresh compost from each drum composter on day 3, 9, 14, and 50 were sampled and stored at −80°C for microbial community analysis.

### 2.3 Physicochemical property analysis of compost

During the composting period, various physicochemical parameters including temperature, pH, electrical conductivity (EC), total carbon (TC), total nitrogen (TN), ammonia nitrogen (NH_4_^+^-N), available phosphorus, emissions of ammonia (NH_3_) and greenhouse gases (CH_4_, CO_2_, and N_2_O) were measured. The fresh compost sample was supplemented with deionized water at a ratio of 1:10 (w/v) and shaken at 200 rpm for 30 min to determine pH and EC using a digital pH meter (S-3C, Leici, China) and a conductivity meter (DDS-307A, Leici, China). TC and TN content of compost were measured by an Elemental analyzer (Thermo Fisher FlashSmart, United States). To determine NH_4_^+^-N content, the sampled compost product was extracted with 2 mol/L KCl solution at solid-to-lipid ratio of 1:5 (w/v). Available phosphorus (AP) content of compost was determined by a continuous flow analysis (SA 4000, Skalar San++, France) after extracting with 0.5 M sodium bicarbonate (NaHCO_3_) (Luo et al., 2024; Wang X. et al., 2023). For the gas collection and analysis, a 50 mL gas sample was collected by a syringe and pulled back three times to ensure the sampled gas homogeneous at each sampling timepoint. Then, the gas sample was transferred into 25 mL pre-evacuated headspace flasks (Vial, crimp, FB, Agilent, United States). During each gas collection, the time and temperature were recorded, and gases were sampled every 10 min. The concentrations of ammonia (NH_3_) and greenhouse gases (CH_4_, CO_2_, and N_2_O) were measured with a gas chromatography (GC, Agilent 7890, United States). After the sampling, fresh air was pump-flushed into the flasks for 5 min to supplement air. Wild soybean (*Glycine soja*), one of representative salt-tolerant halophytes widely distributed in the Yellow River Delta, China (Yin et al., 2023), was used as the tested plant to evaluate the promotion efficiency of tail vegetable compost as a seed germination agent. Seed germination index (GI) was calculated as follows:


$$GI\;\left(\%\right)=\frac{\begin{array}{l} \mathrm{Seed germination rate of}\;\\ \mathrm{treatment}\;\left(\%\right)\times \mathrm{Root length of treatment}\; \end{array}}{\begin{array}{l} \mathrm{Seed germination rate of}\;\\ \mathrm{control}\;\left(\%\right)\times \mathrm{Root length of control} \end{array}}\;\times 100\%$$

### 2.4 Microbial analysis of compost

To investigate the effect of tobacco leaf amendments on the diversity and composition of compost bacterial community, PCR amplification of 16S rRNA high-throughput sequencing analysis was conducted. Total DNA was extracted from 0.5 g of fresh compost using the FastDNA SPIN Kit (MP Biomedicals, Santa Ana, CA, United States) according to manufacturer’s instructions. DNA quality and quantity were determined using a NanoDrop 2000 UV–vis spectrophotometer (Thermo Scientiffc, Wilmington, United States), and 1% agarose gel electrophoresis, respectively. The V3–V4 region of 16S rRNA genes was amplified using the primers 338F (5′-barcode-ACTCCTACGGGAGGCAGCAG-3′) and 806R (5′-GGACTACHVGGGTWTCTAAT-3′). The PCR products were purified and quantified, subsequently, library was constructed and sequenced at the MiSeq^®^ Illumina 2000 platform by Majorbio Bio-Pharm Technology Co., Ltd. (Shanghai, China) (Wang X. et al., 2023). The sequencing reads were analyzed by QIIME (version 2.0.0). Operational taxonomic units (OTUs) were clustered with 97% identify. Taxonomic classification of each OTU was conducted using the RDP classifier against the SILVA database. PICRUSt 2 was used for functional profile prediction of bacterial 16S rRNA based on the Kyoto Encyclopedia of Genes and Genomes (KEGG) database (Wang et al., 2024).

### 2.5 Statistical analysis

All the data were expressed as mean values. Each treatment was replicated three times. Statistical analyses were conducted using the Statistical Product and Service Solutions Software 22 (SPSS Inc., Chicago, United States). Significant differences among different treatments were analyzed by the one-way analysis of variance (ANOVA) using the Duncan’s multiple-comparison test (*p* < 0.05). Prior to the ANOVA analysis, the normality and homogeneity of data was assessed by the Shapiro–Wilk tests and Levene test, respectively. All the data were analyzed using the SPSS Statistics 20.0 at a “*p* < 0.05” level. The OTU richness and Shannon diversity index of bacterial community were determined using the R vegan package. Nonmetric multidimensional scaling (NMDS) analysis was conducted to visualize the similarity of bacterial community structures among different treatments.

## 3 Results and discussion

### 3.1 Changes in the physicochemical properties of compost

**Figure 1:**
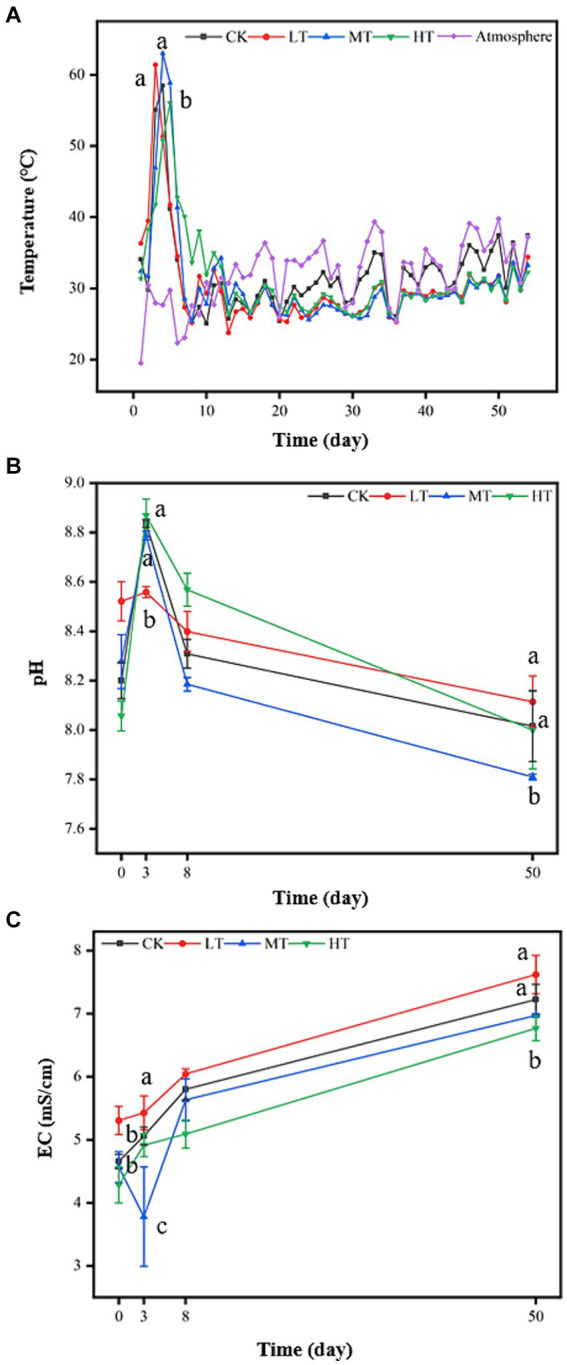
Effects of tobacco leaf amendments of basic physicochemical properties of tomato tail vegetable composting; **(A)** temperature, **(B)** pH and **(C)** electrical conductivity. CK: tomato tail vegetable + cattle manure + rice husk; LT: tomato tail vegetable + cattle manure + rice husk + 2% (w/w) tobacco leaves; MT: tomato tail vegetable + cattle manure + rice husk + 5% (w/w) tobacco leaves; HT: tomato tail vegetable + cattle manure + rice husk + 10% (w/w) tobacco leaves. The different lowercase letters on day 3 and 50 represent significant difference between different treatments (Duncan’s multiple-comparison test, *p* < 0.05).

The four treatment groups went through three typical temperature change stages (mesophilic, thermophilic, and cooling) during the aerobic composting (Figure 1A). Temperature was a crucial indicating parameter of composting, directly reflecting composting progress, maturation extent, and microbial growth and metabolic activities (Kong et al., 2022; Sun et al., 2022). The similar change trends of temperature during composting were found in all treatments, which contained heating, thermophilic, cooling and maturity stages (Figure 1A). Due to rapid decomposition of organic matter by microbes (exothermic reaction), the temperature of compost increased sharply to 50°C–60°C at the beginning stage (day 5–7) of composting in all the treatments. Compared with CK treatment, medium and high-dosed amendments of tobacco leaf generally lengthened the thermophilic stage of composting to 1–2 days, following HT > MT. However, the low-dosed addition of tobacco leaves had little effect on the duration time of thermophilic stage (Figure 1A). While for the peak temperature of composting, MT treatment occupied the highest temperature (63°C), followed by LT treatment (62°C) and CK (59°C). The results showed that tobacco leaf amendment accelerated the compost degradation at the thermophilic stage. In this stage, the anaerobic microorganisms and aerobic microorganisms could efficiently participate in the decomposition and transformation of organic matter at 40°C–60°C (Huang et al., 2022; Li et al., 2023). Therefore, the increased compost temperature at the thermophilic stage could accelerate the decomposition rate of organic matter in the compost. This could speed up the composting process and shorten the composting cycle, elevating the compost quality (Sun et al., 2022). Additionally, the high temperature (>50°C) can kill the pathogenic microorganisms and decompose toxic substances in the compost efficaciously, increasing the environmental safety of composting products (Huang et al., 2022). After the thermophilic stage, readily-decomposed organic matter and available carbon sources are gradually exhausted with the decreasing microbial activity, so the temperature gradually decreased (Li et al., 2023). Notably, MT and HT treatments put off the decrease of temperature, but LT had little impact on it, exhibiting the dose-dependent effect. The effects of tobacco leaf amendments on compost temperature could be ascribed to that the supply of N-containing nutrients from tobacco leaves promoted microbial metabolism and accelerated the organic matter degradation, resulting in the generation of more heat (Kurt and Kinay, 2021; Li et al., 2023).

The pH was an indicator of compost maturity, which affected many biological processes, including the degradation of organic matter. Variation of pH during composting was related with ammonification, nitrification, NH_3_emission and microbe-mediated N transformation. During composting, the pH increased gradually and reached the peak at day 3, LT treatment decreased the compost pH, while MT and HT treatments did not show the similar effect on pH (Figure 1B). The compost pH then decreased from day 3 to 50. This was ascribed to volatilization of NH_3_, emission of CO_2_ and production of organic acids (Wong et al., 2017). Moreover, the pH was highest in HT treatment during composting on day 8. This result is probably because high-dosed amount addition of tobacco leaves promoted the decomposition of organic nitrogen compounds and the production of ammonia as a result of promoted microbial activities (Huang et al., 2022; Li et al., 2023), strongly agreed with the higher compost temperature after HT treatment (Figure 1A). At the end of composting, pH in all the treatments was 7.8–8.2, which was within the required standards (5.5–8.5) of the Chinese Industry Standard (Wang N. et al., 2022). The EC reflected the degradation of organic matter, which could be used to evaluate the supply of nutrient elements in the compost (Wang et al., 2021). The EC values in all treatments increased at the inception stage of the composting (Figure 1C). This was mainly attributed to microbial decomposition of organic matter, which produced the large number of small molecular fatty acids and other small molecular substances (Wang et al., 2021; Xi et al., 2016). However, in the initial phase of composting, the EC values of compost treated with MT were significantly lower by 7.69% than that of control (Figure 1C) due to the precipitation and adsorption of mineral salts by the lignocellulose resulted from the increased compost pH after the addition of tobacco leaves (Li et al., 2023; Wang G. et al., 2022).

### 3.2 Nutrient condition of compost

**Figure 2:**
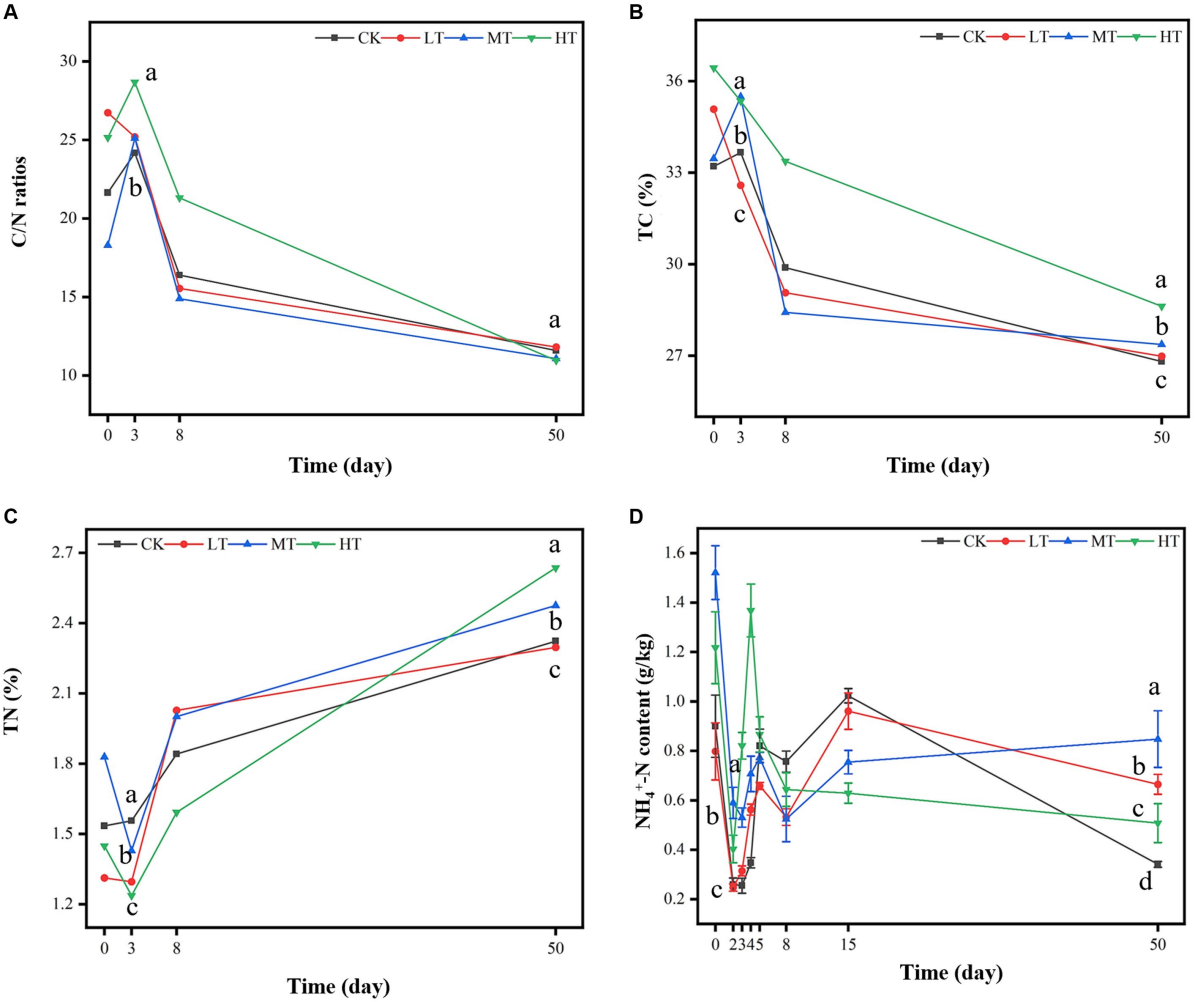
Effects of tobacco leaf amendments of nutrient parameters of tomato tail vegetable composting; **(A)** C/N ratios, **(B)** total carbon content (TC) content, **(C)** total nitrogen (TN) content, **(D)** ammonia nitrogen (NH_4_^+^-N) content. CK: tomato tail vegetable + cattle manure + rice husk; LT: tomato tail vegetable + cattle manure + rice husk + 2% (w/w) tobacco leaves; MT: tomato tail vegetable + cattle manure + rice husk + 5% (w/w) tobacco leaves; HT: tomato tail vegetable + cattle manure + rice husk + 10% (w/w) tobacco leaves. The different lowercase letters on day 3 and 50 represent significant difference between different treatments (Duncan’s multiple-comparison test, *p* < 0.05).

The C/N ratios reflected the maturation degree of compost (Chen et al., 2021). With the extension of composting time, the C/N ratios increased sharply by 130–300% in all the treatments possibly due to rapid decomposition of nitrogenous compounds by microbes and emission of NH_3_ at the thermophilic stage (Figure 2A). On day 3 and 8, HT treatment increased the C/N ratios of compost compared with CK treatment. This could be ascribed to input of high-amount of N-containing in tobacco leaves to compost (Wang G. et al., 2022). However, tobacco amendments had little effect on C/N ratios. After the thermophilic stage of composting, the C/N ratios in all the treatments decreased and eventually were under 20, which meet the standard of compost maturity (Chen et al., 2021). After day 3, the microbial activity was enhanced, which could lead to the increased consumption of carbon-containing substrates (e.g., sugars and organic acids) in the compost, thereby resulting in the decrease of compost TC content (Figure 2B). However, till to the ending of composting, the TC content of compost amended with tobacco leaves was significantly higher than CK. However, the TN content exhibited the increasing tread as the time longed (Figure 2C), perhaps owing to the mass reduction (Li et al., 2023). Relative to CK treatment, the TN content of the final composting product in HT, MT and LT treatments were increased by 11.40, 20.44, and 34.35%, respectively (Figure 2A).

NH_4_^+^-N content in all treatments firstly decreased from day 1 to 2 and subsequently increased from day 2 to 4 (Figure 2D). The increase of NH_4_^+^-N concentration in the early stage of composting may be due to the decomposition of organic matter and ammoniation of organic N by microorganisms (Wang G. et al., 2022). Compared with CK treatment, tobacco leaf amendments increased NH_4_^+^-N content of compost on day 3 and 4, with the order of HT (1.39 g/kg) > MT (0.70 g/kg) > LT (0.30 g/kg), exhibiting the obvious dose-dependent effect (Figure 2D). On day 50, the NH_4_^+^-N content of MT (0.80 g/kg), LT (0.70 g/kg) and HT (0.57 g/kg) were generally higher than that of CK treatment, and met the mature requirements of compost (more than 400.0 mg/kg) (Zhang et al., 2021). This result indicated that N-containing organic matter and mineral salts in tobacco leaves was converted into inorganic N during the thermophilic stage of composting. Notably, TN and NH_4_^+^-N content of compost in tobacco leaf treatments were generally higher than that of CK (Figure 2D), indicating that tobacco leaf amendments enhanced the N retention in compost product (Li et al., 2023).

### 3.3 Change of bacterial community diversity and structure in the compost

**Figure 3:**
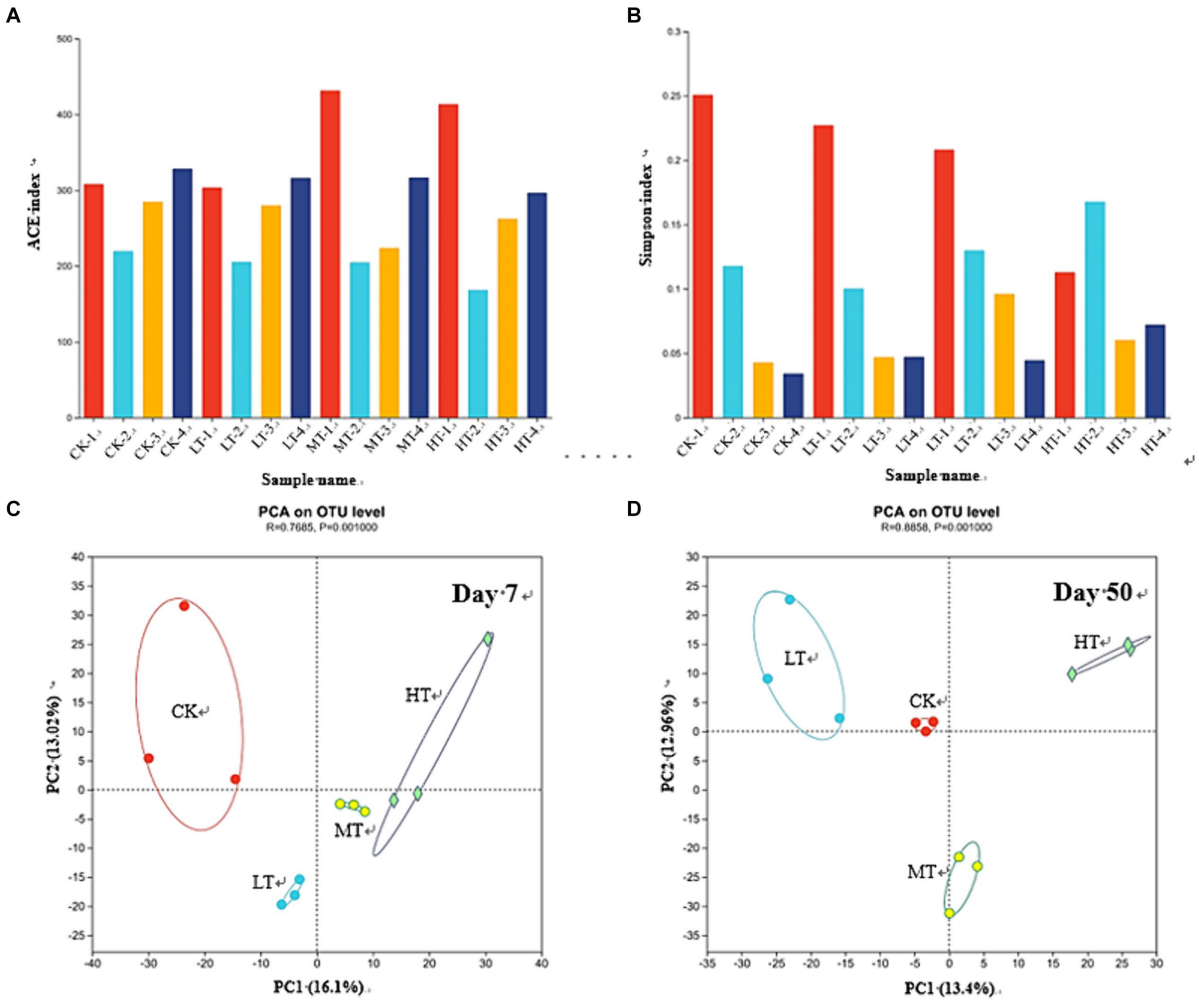
Effects of tobacco leaf amendments of bacterial community diversity and structure in the compost with or without tobacco leaf amendments. **(A)** The richness index of bacterial community (ACE index). **(B)** The diversity index of bacterial community (Shannon index). **(C)** Principal component analysis (PCA) of bacterial community composition according to the Bray–Curtis similarity at day 7. **(D)** PCA of bacterial community composition according to the Bray–Curtis similarity at day 50. CK: tomato tail vegetable + cattle manure + rice husk; LT: tomato tail vegetable + cattle manure + rice husk + 2% (w/w) tobacco leaves; MT: tomato tail vegetable + cattle manure + rice husk + 5% (w/w) tobacco leaves; HT: tomato tail vegetable + cattle manure + rice husk + 10% (w/w) tobacco leaves. -1, -2, -3, and -4 in the panels **A**,**B** represented the samples collected from the compost on day 1, 3, 7, and 50, respectively.

Tobacco leaf amendments affected bacterial α-diversity of compost during the different stages of composting, which was evaluated based on ACE richness and Simpson diversity index (Figures 3A,B). For the ACE index, MT and HT treatments increased it in the compost on day 1. However, the ACE index underwent a sharp decrease on day 3 relative to it on day 1 (Figure 3A). This could be ascribed to the non-suitable microenvironment (e.g., oxygen and labile nutrient deficiency) of compost for microbial growth at the initial stage of composting (Qian et al., 2022). Similar results were also observed for Simpson index (Figure 3B). As the composting process progressed, the microorganisms gradually adapted and grew, resulting in the increased microbial diversity (Chen et al., 2022). Moreover, tobacco leaf amendments had non-significant effect on the ACE index on day 3, 7 and 50. These results indicated that amendments of tobacco leaves did not increase the number of bacterial species in the composting system. MT and HT treatments elevated the Simpon index of composting on day 3 and 50 (Figure 3B), which could be resulted from the input of nutrients (e.g., N-containing organic matter and mineral salts) from tobacco leaves into the compost promoted microbial metabolism and accelerated the organic matter degradation (Kurt and Kinay, 2021; Li et al., 2023).

The Bray–Curtis based PCA (Figures 3C,D) showed that tobacco leaves changed the bacterial community composition. During the thermophilic stage, the tobacco leaf amendments remarkably changed the bacterial community composition of compost compared with CK treatment, revealed by the distinct clustering features between CK and tobacco leaf treatments (LT, MT, and HT). Among the tobacco leaf treatments, the MT treatment was located close to HT treatment (Figure 3C). However, at the ending of composting on day 50, every tobacco leaf treatment moved far from CK treatment, showing distinctly different clustering features of bacterial community between each other (Figure 3D). These results indicated that susceptibility of bacterial community of compost to different doses of tobacco leaves changed with different composting stages.

### 3.4 Change of bacterial community composition in the compost

**Figure 4:**
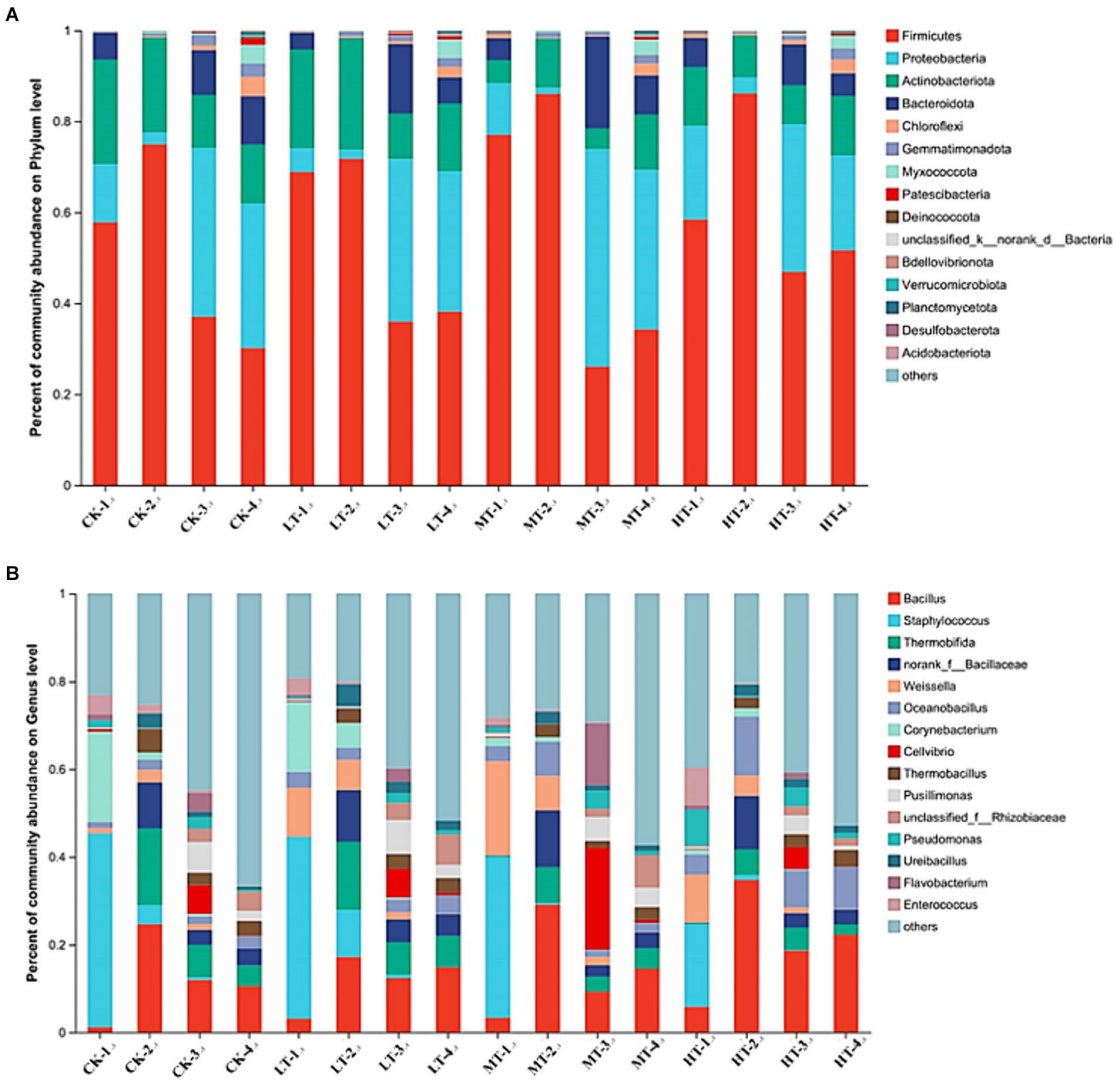
Profiles of bacterial community composition in the compost with or without tobacco leaf amendments on day 50. **(A)** Cumulative histogram showing the relative abundance of top 15 bacterial phyla. **(B)** Cumulative histogram showing the relative abundance of top 30 bacterial genera. CK: tomato tail vegetable + cattle manure + rice husk; LT: tomato tail vegetable + cattle manure + rice husk + 2% (w/w) tobacco leaves; MT: tomato tail vegetable + cattle manure + rice husk + 5% (w/w) tobacco leaves; HT: tomato tail vegetable + cattle manure + rice husk + 10% (w/w) tobacco leaves. -1, -2, -3, and -4 in the panels **A,B** represented the samples collected from the compost on day 1, 3, 7, and 50, respectively.

The bacterial community in the composting system at different compost stage were analyzed using 16S rRNA high-throughput sequencing. Figures 4A,B showed the changes in bacterial abundance at the phylum level and the top 30 bacterial genera. Firmicutes, Proteobacteria, Actinobacteria, Bacteroidota, Chloroflexi, and Gemmatimonadota were the dominant bacteria throughout the entire aerobic composting process. These bacterial phyla accounted for 85.08–98.35% of the total bacterial 16S rRNA gene sequences (Figure 4A). The bacterial community was dominated by Firmicutes at the initial stage of composting (day 1 and 3), although the proportion varied among different treatments (35.4%–87.5%). During the thermophilic period, the relative abundance of Firmicutes in the MT and HT group increased relative to the CK (CK-1 and CK-2) group. Proteobacteria and Bacteroidota were enriched in the higher-doses of tobacco leaf treatments (MT-1, -2 and HT-1, -2) at the initial stage of thermophilic period compared with the CK group. However, at the peak of thermophilic period (day 7), LT-3 treatment showed little effect on bacterial community at the phylum level relative to CK, despite the relative abundance of Proteobacteria and Firmicutes increased in the MT-3 and HT-3 treatments (Figure 4A). It was reported that some members of phylum Bacteroidota and Firmicutes could degrade lignocellulosic polymers, which can be fermented into short chain volatile fatty acids (Wang C. et al., 2022). Actinobacteriota could survive in tough environments, such as high temperatures, effectively degrade organic matter and lignocelluloses (He J. et al., 2022; Zhao et al., 2016). Proteobacteria was associated with compost nitrogen transformation and was dominant during thermophilic and cooling periods. Proteobacteria, recognized as N-fixing bacteria, could be beneficial for mitigating N loss and N retention in the compost (Wang Z. et al., 2023). Therefore, the tobacco leaf induced response changes of bacterial community in the compost could contribute to increasing the compost maturity and N retention.

The taxonomic composition during composting at the genus level was also investigated (Figure 4B). In the CK treatment, the dominant genera in the initial stage of composting were *Staphylococcus* and *Corynebacterium*. However, after tobacco leaf amendments, the compost was dominantly occupied by *Staphylococcus* and *Weissella*. At the thermophilic stage, the relative abundance of *Bacillaceae*, *Weissella* and *Cellvibrio* generally increased after the tobacco leaf amendments in the MT-2/3 and HT-2/3 treated composts compared with the CK treatment (Figure 4B). As a member of Firmicutes, *Bacillaceae* is a typical sporulating and heat resist species (Wang C. et al., 2022). Many other functional bacteria, such as *Weissella*, a thermophilic bacterium that can hydrolyze phenolic compounds, existing with *Bacillaceae*, improving the community stability (Wang et al., 2019). A previous study also found that *Luteimonas*, *Cellvibrio* and *Pseudomonas* were the main bacterial genera in the maturation stage of composting (Yin et al., 2021). *Cellvibrio*, a cellulose-degrading bacteria with low tolerance to high temperature (Wang Z. et al., 2022), were found to be more abundant in the late stage of compost in the MT and HT treatments, indicating that tobacco leaf amendments facilitated the compost humification.

### 3.5 Analysis of functional genes related to nitrogen transformation in the compost

**Figure 5:**
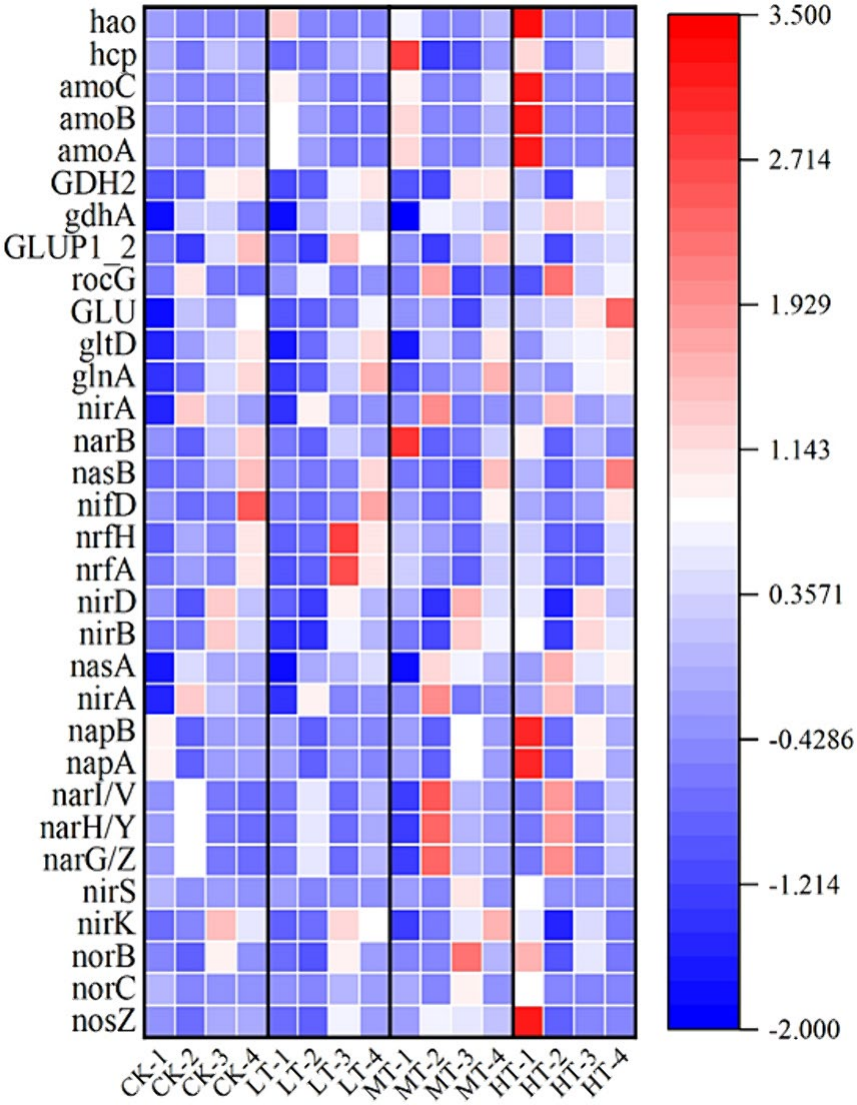
Heatmap showing expression profiles of N-transformation functional genes in the compost. CK: tomato tail vegetable + cattle manure + rice husk; LT: tomato tail vegetable + cattle manure + rice husk + 2% (w/w) tobacco leaves; MT: tomato tail vegetable + cattle manure + rice husk + 5% (w/w) tobacco leaves; HT: tomato tail vegetable + cattle manure + rice husk + 10% (w/w) tobacco leaves. -1, -2, -3, and -4 represented composted which was collected on day 1, 3, 7, and 50, respectively. Color intensity of the scale indicated the relative abundance of each gene based on the log2 (fold change) values.

The bacterial metabolic function prediction was carried out using the PICRUSt 2 based on KEGG pathway database. The expression profiles of N conversion-associated genes were further investigated to elucidate the mechanism of N transformation in the compost (Figure 5). As for nitrification process of compost, *amoA*, *amoB*, and *amoC* are three key genes encoding subunits of ammonia monooxygenase, whose relative abundance was generally higher in tobacco leaf groups than in CK group. Moreover, the promotion effect followed the order of HT > MT > LT, showing the dose-dependent effect. This result suggested tobacco leaf amendments elevated the activity and abundance of ammonia oxidizing bacteria, which could be resulted from changes of environmental factors such as composting temperature and oxygen content (Xiong et al., 2022). Similarly, the relative abundance of hydroxylamine oxidase (*hao*) gene in the tobacco leaf treatments was higher than CK treatments. Taken together, the addition of tobacco leaves promoted the nitrification process, converting more NH_4_^+^-N to NO_3_^−^-N. However, as the composting time going, the relative abundance of ammonia oxidizing genes exhibited no significant difference in tobacco leaf treatments from CK treatment, implying that ammonia oxidation in compost was inhibited possibly due to the decreased activity and abundance of nitrifying bacteria after thermophilic and maturation phases (Figure 5).

For the denitrifying bacterial genes such as *nirA*, *nirS* and *nirK*, the tobacco leaf amendments elevated their relative abundance at the early stage, but decreased their relative abundance during the thermophilic and maturation phases. The different effect of tobacco leaf amendments on denitrifying bacterial gene at different composting stages could be explained by biodegradation extent and O_2_ content (Zhong et al., 2020). At the early stages of composting, strong biodegradation occurred and O_2_ was rapidly consumed, providing a favorable anaerobic environment for denitrifying bacteria (Tian et al., 2024; Zhong et al., 2020). With the constant consumption of easily degradable organic matter in the compost, the composting was gradually stabilized (Xiong et al., 2022). These could explain the variable responses of bacterial genes in the compost to tobacco leaf amendments at the different composting stages (Figure 5). The *norB* gene was responsible for the denitrification step in the conversion of NO into N_2_O. A significantly higher *norB* gene relative abundance was observed on day 1 and 7 in tobacco leaf treatments than CK, but the difference became smaller as the composting proceeded except for the MT-3 treatment. The relative abundance of *nosZ* gene in the HT-1 treatment was higher than that in the CK-1 treatment. However, the relative abundance of *nosZ* gene remained constant in individual tobacco leaf treatment during the thermophilic and maturation phases. The reason behind this observation might be that the *nosZ* gene is associated with bacteria with an extreme tolerance to high temperatures. These results strongly agreed with a previous study showing that the temperature was one of the main factors affecting the denitrifier bacterial community in the compost (Zhou et al., 2022).

### 3.6 Evaluation of tail vegetable compost as a seed germination agent for halophyte

**Figure 6:**
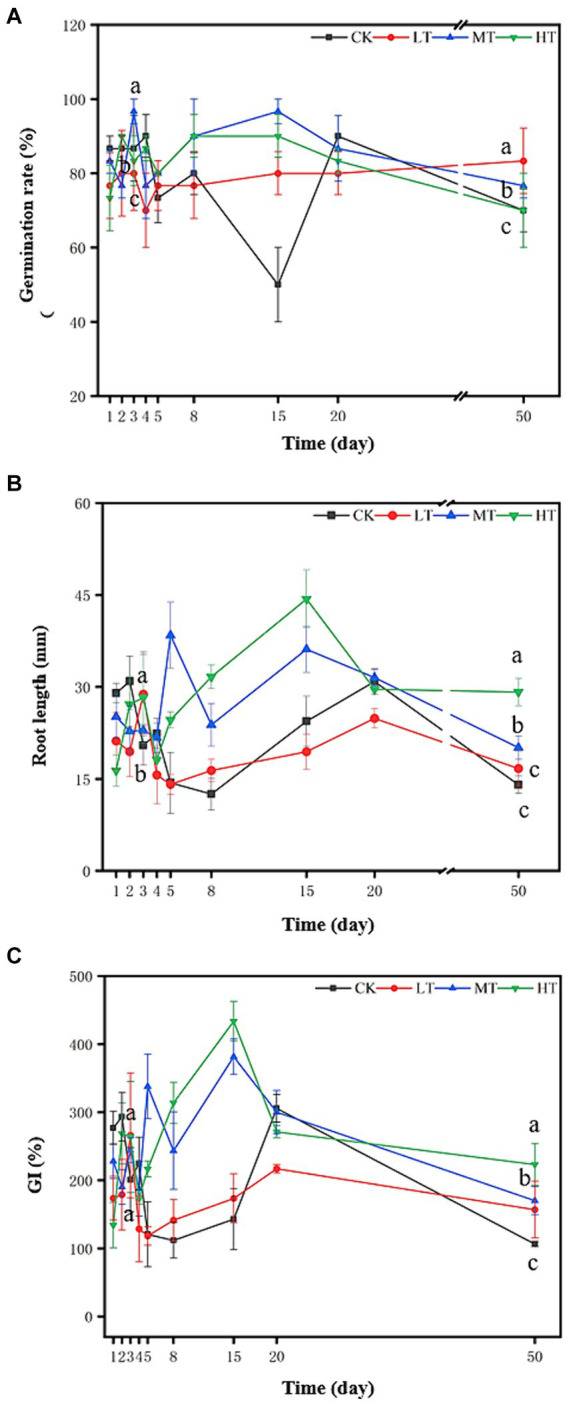
Effect of tail vegetable compost leachate on the seed germination of wild soybean (*Glycine soja*). **(A)** Germination rate; **(B)** root length and **(C)** germination index (GI). CK: tomato tail vegetable + cattle manure + rice husk; LT: tomato tail vegetable + cattle manure + rice husk + 2% (w/w) tobacco leaves; MT: tomato tail vegetable + cattle manure + rice husk + 5% (w/w) tobacco leaves; HT: tomato tail vegetable + cattle manure + rice husk + 10% (w/w) tobacco leaves. The different lowercase letters on day 3 and 50 represent significant difference between different treatments (Duncan’s multiple-comparison test, *p* < 0.05).

The composting leachate in all the treatments was also prepared to evaluate the effect of compost products on seed germination and root development of local halophyte, i.e., wild soybean (*Glycine soja*). On day 3, the germination rate of wild soybean in the MT treatment was significantly higher than those in other treatments (Figure 6A). Similarly, all the tobacco leaf treatments occupied the obviously higher seed germination rate compared with CK at the thermophilic and cooling periods (from day 8 to 15), following the order of MT > HT > LT. While the seed germination rate in the LT treatment was higher than that of CK treatment, but no similar results were found in other tobacco leaf treatments (Figure 6A). These results showed that tobacco leaf amendments elevated the efficiency of composting products in promoting seed germination of halophyte. For the root length, the higher doses of tobacco leaf amendments (MT and HT treatments) increased it, with the order of MT > HT, but the root length of wild soybean in the LT treatment exhibited non-significant difference from that in the CK (Figure 6B). At the cooling and ending stages (from day 15 to 50) of composting, tobacco leaf amendments generally increased the root length of wild soybean relative to CK, following HT (101%) > MT (33.4%) > LT (13.3%). The result suggested the dose-dependent promotion effect of tobacco leaf amendments on seed germination and root development of halophyte. This can be explained by the supply of water soluble N-containing nutrients from tobacco leaf treated composts (Yuan et al., 2019).

The germination index (GI) can be used to assess the phytotoxicity and maturity of compost (Yang et al., 2021). When the GI is lower than 50.0%, there are more toxic substances in the heap, which has an inhibitory effect on plant growth. When the GI is greater than 70.0%, the heap is decomposed and non-toxic, and can be used for fertilizer application, which is conducive to plant growth and development (Wang G. et al., 2022). As shown in Figure 6C, GI index gradually increased with the composting processed in all the four treatments. On day 5, all the compost treatments reached the maturity standard (80%). Compared with CK treatment, the GI in the MT and HT treatments were significantly higher on day 8 and 15, showing an obvious enhancement on the compost maturity (Figure 6C). This could be attributed to the higher nutrient content and temperature in tobacco leaf treated compost. When the final composts were subjected to phytotoxicity tests on day 50, the GI were significantly higher in the treatments with tobacco leaf additives than in CK, but did not differ significantly among the three treatments (Figure 6C). Seed germination is inhibited by phytotoxic substances such as low molecular weight organic acids, heavy metals, and inorganic nitrogen in the aqueous extracts of composts (Chen et al., 2021). Composting can decrease phytotoxicity by degradation, transformation, thereby reducing toxin bioavailability (Li et al., 2023; Wang G. et al., 2022). Therefore, the increased GI in the compost after tobacco leaf treatment could be resulted from the higher peak temperature and increased nutrient supply in the compost with tobacco leaf (Figure 1A), which can effectively kill the pathogenic microorganisms and elevate the compost quality.

## 4 Conclusion

Resource utilization of tail vegetables has become an urgent issue to be addressed in the modern agriculture. In the present study, the effect of different doses of tobacco leaf amendments on the N transformation, compost quality and microbial regulation of tomato tail vegetable composting was firstly investigated. The results showed that high-doses (5% w/w and 10% w/w) of tobacco leaves elevated the maturity of compost, enhanced N conservation, changed bacterial community structure during the composting. Moreover, owing to supply of N-containing nutrients from tobacco leaves and promoted organic matter degradation, the population diversity and N-related microbial metabolism (i.e., hydroxylamine oxidase and denitrifying bacterial gene) of compost was enhanced after flue-cured tobacco leaf amendments. Besides, tobacco leaf amendments promoted the seed germination and root development of wild soybean, representative of halophyte. These findings showed the great potential of flue-cured tobacco leaves as additives during the aerobiotic composting of tail vegetables. Furthermore, tobacco leaf amendments increased the efficiency of composting product into promoting salt-tolerant plant growth and elevating the primary productivity of salt-affected soils. Therefore, it could be a promising field remediation method to enhance the soil health and primary productivity for the salt-affected wetland ecosystems using the tobacco-modified composting products. Considering the significant role of tobacco leaf-modified compost properties, quality and induced microbial N transformation responses, it could be a good choice to screen and inoculate N-transforming functional bacterial taxa with higher performance in stimulating ammonifying process to further enhance labile N retention and availability in the compost.

## Data Availability

The raw data supporting the conclusions of this article will be made available by the authors, without undue reservation.

## References

[ref1] Ba S. Qu Q. Zhang K. Groot J. C. J. (2020). Meta-analysis of greenhouse gas and ammonia emissions from dairy manure composting. Biosyst. Eng. 193, 126–137. doi: 10.1016/j.biosystemseng.2020.02.015

[ref2] Chen Z. Fu Q. Cao Y. Wen Q. Wu Y. (2021). Effects of lime amendment on the organic substances changes, antibiotics removal, and heavy metals speciation transformation during swine manure composting. Chemosphere 262:128342. doi: 10.1016/j.chemosphere.2020.128342 33182112

[ref3] Chen Y. Tang P. Li Y. Chen L. Jiang H. Liu Y.. (2022). Effect of attapulgite on heavy metals passivation and microbial community during co-composting of river sediment with agricultural wastes. Chemosphere 299:134347. doi: 10.1016/j.chemosphere.2022.134347, PMID: 35306052

[ref4] He X. Zhang T. Niu Y. Xue Q. Ali E. F. Shaheen S. M.. (2022). Impact of catalytic hydrothermal treatment and Ca/Al-modified hydrochar on lability, sorption, and speciation of phosphorus in swine manure: microscopic and spectroscopic investigations. Environ. Pollut. 299:118877. doi: 10.1016/j.envpol.2022.118877, PMID: 35077837

[ref5] He J. Zhu N. Xu Y. Wang L. Zheng J. Li X. (2022). The microbial mechanisms of enhanced humification by inoculation with *Phanerochaete chrysosporium* and *Trichoderma longibrachiatum* during biogas residues composting. Bioresour. Technol. 351:126973. doi: 10.1016/j.biortech.2022.126973, PMID: 35292388

[ref6] Hoang H. G. Thuy B. T. P. Lin C. Vo D.-V. N. Tran H. T. Bahari M. B.. (2022). The nitrogen cycle and mitigation strategies for nitrogen loss during organic waste composting: a review. Chemosphere 300:134514. doi: 10.1016/j.chemosphere.2022.134514, PMID: 35398076

[ref7] Huang D. Gao L. Cheng M. Yan M. Zhang G. Chen S.. (2022). Carbon and N conservation during composting: a review. Sci. Total Environ. 840:156355. doi: 10.1016/j.scitotenv.2022.156355, PMID: 35654189

[ref8] Jin S. Zhang B. Wu B. Han D. Hu Y. Ren C.. (2021). Decoupling livestock and crop production at the household level in China. Nat. Sustain. 4, 48–55. doi: 10.1038/s41893-020-00596-0

[ref9] Kong Y. Ma R. Li G. Wang G. Liu Y. Yuan J. (2022). Impact of biochar, calcium magnesium phosphate fertilizer and spent mushroom substrate on humification and heavy metal passivation during composting. Sci. Total Environ. 824:153755. doi: 10.1016/j.scitotenv.2022.153755, PMID: 35151730

[ref10] Kurt D. Kinay A. (2021). Effects of irrigation, nitrogen forms and topping on sun cured tobacco. Ind. Crop. Prod. 162:113276. doi: 10.1016/j.indcrop.2021.113276

[ref11] Li D. Manu M. K. Varjani S. Wong J. W. C. (2023). Role of tobacco and bamboo biochar on food waste digestate co-composting: nitrogen conservation, greenhouse gas emissions, and compost quality. Waste Manag. 156, 44–54. doi: 10.1016/j.wasman.2022.10.022, PMID: 36436407

[ref12] Lisuma J. Mbega E. Ndakidemi P. (2020). Influence of tobacco plant on macronutrient levels in sandy soils. Agronomy 10:418. doi: 10.3390/agronomy10030418

[ref13] Luo X. Chen W. Liu Q. Wang X. Miao J. Liu L.. (2024). Corn straw biochar addition elevated phosphorus availability in a coastal salt-affected soil under the conditions of different halophyte litter input and moisture contents. Sci. Total Environ. 908:168355. doi: 10.1016/j.scitotenv.2023.168355, PMID: 37952652

[ref14] Qian X. Bi X. Xu Y. Yang Z. Wei T. Xi M.. (2022). Variation in community structure and network characteristics of spent mushroom substrate (SMS) compost microbiota driven by time and environmental conditions. Bioresour. Technol. 364:127915. doi: 10.1016/j.biortech.2022.127915, PMID: 36089128

[ref15] Qiao Y. Tie J. Wang X. Wei B. Zhang W. Liu Z.. (2023). Comprehensive evaluation on effect of planting and breeding waste composts on the yield, nutrient utilization, and soil environment of baby cabbage. J. Environ. Manag. 341:117941. doi: 10.1016/j.jenvman.2023.117941 37178544

[ref16] Shan G. Li W. Gao Y. Tan W. Xi B. (2021). Additives for reducing nitrogen loss during composting: a review. J. Clean. Prod. 307:127308. doi: 10.1016/j.jclepro.2021.127308

[ref17] Sun S. Abdellah Y. A. Y. Miao L. Wu B. Ma T. Wang Y.. (2022). Impact of microbial inoculants combined with humic acid on the fate of estrogens during pig manure composting under low-temperature conditions. J. Hazard. Mater. 424:127713. doi: 10.1016/j.jhazmat.2021.127713, PMID: 34815123

[ref18] Tian X. Qin W. Zhang Y. Liu Y. Lyu Q. Chen G.. (2024). The inoculation of thermophilic heterotrophic nitrifiers improved the efficiency and reduced ammonia emission during sewage sludge composting. Chem. Eng. J. 479:147237. doi: 10.1016/j.cej.2023.147237

[ref19] Wang X. Kong Q. Cheng Y. Xie C. Yuan Y. Zheng H.. (2024). Cattle manure hydrochar posed a higher efficiency in elevating tomato productivity and decreasing greenhouse gas emissions than plant straw hydrochar in a coastal soil. Sci. Total Environ. 912:168749. doi: 10.1016/j.scitotenv.2023.168749, PMID: 38007120

[ref20] Wang G. Kong Y. Liu Y. Li D. Zhang X. Yuan J.. (2020). Evolution of phytotoxicity during the active phase of co-composting of chicken manure, tobacco powder and mushroom substrate. Waste Manag. 114, 25–32. doi: 10.1016/j.wasman.2020.06.034, PMID: 32645612

[ref21] Wang X. Li Z. Cheng Y. Yao H. Li H. You X.. (2023). Wheat straw hydrochar induced negative priming effect on carbon decomposition in a coastal soil. iMeta 2:e134. doi: 10.1002/imt2.134, PMID: 38868226 PMC10989761

[ref22] Wang M. Liu Y. Wang S. Wang K. Zhang Y. (2021). Development of a compound microbial agent beneficial to the composting of Chinese medicinal herbal residues. Bioresour. Technol. 330:124948. doi: 10.1016/j.biortech.2021.124948, PMID: 33735731

[ref23] Wang J. Liu Z. Xia J. Chen Y. (2019). Effect of microbial inoculation on physicochemical properties and bacterial community structure of citrus peel composting. Bioresour. Technol. 291:121843. doi: 10.1016/j.biortech.2019.121843, PMID: 31357046

[ref24] Wang C. Wu M. Peng C. Yan F. Jia Y. Li X.. (2022). Bacterial dynamics and functions driven by a novel microbial agent to promote kitchen waste composting and reduce environmental burden. J. Clean. Prod. 337:130491. doi: 10.1016/j.jclepro.2022.130491

[ref25] Wang Z. Xu Y. Yang T. Liu Y. Zheng T. Zheng C. (2023). Effects of biochar carried microbial agent on compost quality, greenhouse gas emission and bacterial community during sheep manure composting. Biochar 5:3. doi: 10.1007/s42773-022-00202-w

[ref26] Wang G. Yang Y. Kong Y. Ma R. Yuan J. Li G. (2022). Key factors affecting seed germination in phytotoxicity tests during sheep manure composting with carbon additives. J. Hazard. Mater. 421:126809. doi: 10.1016/j.jhazmat.2021.126809, PMID: 34388932

[ref27] Wang N. Zhao K. Li F. Peng H. Lu Y. Zhang L.. (2022). Characteristics of carbon, nitrogen, phosphorus and sulfur cycling genes, microbial community metabolism and key influencing factors during composting process supplemented with biochar and biogas residue. Bioresour. Technol. 366:128224. doi: 10.1016/j.biortech.2022.128224, PMID: 36328174

[ref28] Wang Z. Zhao M. Xie J. Wang Z. Tsui T.-H. Ren X.. (2022). Insight into the fraction variations of selenium and their effects on humification during composting. Bioresour. Technol. 364:128050. doi: 10.1016/j.biortech.2022.128050, PMID: 36184014

[ref29] Wong J. W. C. Karthikeyan O. P. Selvam A. (2017). Biological nutrient transformation during composting of pig manure and paper waste. Environ. Technol. 38, 754–761. doi: 10.1080/09593330.2016.1211747, PMID: 27448944

[ref30] Xi B. Zhao X. He X. Huang C. Tan W. Gao R.. (2016). Successions and diversity of humic-reducing microorganisms and their association with physical-chemical parameters during composting. Bioresour. Technol. 219, 204–211. doi: 10.1016/j.biortech.2016.07.120, PMID: 27494101

[ref31] Xie S. Tran H.-T. Pu M. Zhang T. (2023). Transformation characteristics of organic matter and phosphorus in composting processes of agricultural organic waste: research trends. Mater. Sci. Energy Technol. 6, 331–342. doi: 10.1016/j.mset.2023.02.006

[ref32] Xiong J. Su Y. He X. Han L. Guo J. Qiao W.. (2022). Effects of functional-membrane covering technique on nitrogen succession during aerobic composting: metabolic pathways, functional enzymes, and functional genes. Bioresour. Technol. 354:127205. doi: 10.1016/j.biortech.2022.127205, PMID: 35462015

[ref33] Yang Y. Wang G. Li G. Ma R. Kong Y. Yuan J. (2021). Selection of sensitive seeds for evaluation of compost maturity with the seed germination index. Waste Manag. 136, 238–243. doi: 10.1016/j.wasman.2021.09.037, PMID: 34700164

[ref34] Ye P. Fang L. Song D. Zhang M. Li R. Awasthi M. K.. (2023). Insights into carbon loss reduction during aerobic composting of organic solid waste: a meta-analysis and comprehensive literature review. Sci. Total Environ. 862:160787. doi: 10.1016/j.scitotenv.2022.160787 36502991

[ref35] Yin Y. Ren Z. Zhang L. Qin L. Chen L. Liu L.. (2023). *In situ* proteomic analysis of herbicide-resistant soybean and hybrid seeds via matrix-assisted laser desorption/ionization-mass spectrometry imaging. J. Agric. Food Chem. 71, 7140–7151. doi: 10.1021/acs.jafc.3c00301, PMID: 37098110

[ref36] Yin Y. Yang C. Tang J. Gu J. Li H. Duan M.. (2021). Bamboo charcoal enhances cellulase and urease activities during chicken manure composting: roles of the bacterial community and metabolic functions. J. Environ. Sci. 108, 84–95. doi: 10.1016/j.jes.2021.02.007, PMID: 34465440

[ref37] Yuan Y. Zou P. Zhou J. Geng Y. Fan J. Clark J.. (2019). Microwave-assisted hydrothermal extraction of non-structural carbohydrates and hemicelluloses from tobacco biomass. Carbohydr. Polym. 223:115043. doi: 10.1016/j.carbpol.2019.115043, PMID: 31426995

[ref38] Zhang Z. Liu D. Qiao Y. Li S. Chen Y. Hu C. (2021). Mitigation of carbon and nitrogen losses during pig manure composting: a meta-analysis. Sci. Total Environ. 783:147103. doi: 10.1016/j.scitotenv.2021.147103, PMID: 34088163

[ref39] Zhao Y. Lu Q. Wei Y. Cui H. Zhang X. Wang X.. (2016). Effect of actinobacteria agent inoculation methods on cellulose degradation during composting based on redundancy analysis. Bioresour. Technol. 219, 196–203. doi: 10.1016/j.biortech.2016.07.117, PMID: 27494100

[ref40] Zheng X. Zou D. Wu Q. Wang H. Li S. Liu F.. (2022). Review on fate and bioavailability of heavy metals during anaerobic digestion and composting of animal manure. Waste Manag. 150, 75–89. doi: 10.1016/j.wasman.2022.06.033, PMID: 35809372

[ref41] Zhong X.-Z. Zeng Y. Wang S.-P. Sun Z.-Y. Tang Y.-Q. Kida K. (2020). Insight into the microbiology of nitrogen cycle in the dairy manure composting process revealed by combining high-throughput sequencing and quantitative PCR. Bioresour. Technol. 301:122760. doi: 10.1016/j.biortech.2020.122760, PMID: 31972401

[ref42] Zhou L. Li J. Pokhrel G. R. Zhao Y. Zhang C. Chu W.. (2022). Effects of monoculture regime on the soil nirK- and nosZ-denitrifying bacterial communities of *Casuarina equisetifolia*. Appl. Soil Ecol. 171:104326. doi: 10.1016/j.apsoil.2021.104326

[ref43] Zittel R. da Silva C. P. Domingues C. E. Seremeta D. C. H. da Cunha K. M. de Campos S. X. (2020). Availability of nutrients, removal of nicotine, heavy metals and pathogens in compounds obtained from smuggled cigarette tobacco compost associated with industrial sewage sludge. Sci. Total Environ. 699:134377. doi: 10.1016/j.scitotenv.2019.134377, PMID: 31671305

